# Severe Enteritis as the Sole Manifestation of Novel Coronavirus Disease 2019 (COVID-19) in Adolescent Patients

**DOI:** 10.1155/2020/8823622

**Published:** 2020-12-23

**Authors:** Sandeep Gupta, Ashlesha Kaushik, Helen Kest, Alexandra K. Charles, William De Bruin, Mario Colletti, David Goldberg

**Affiliations:** ^1^Division of Pulmonary and Critical Care, Unity Point Health at St. Luke's Regional Medical Center, 2720 Stone Park Blvd, Sioux City, Iowa 51104, USA; ^2^Pediatric Infectious Diseases, Unity Point Health at St. Luke's Regional Medical Center and University of Iowa Carver College of Medicine, 2720 Stone Park Blvd, Sioux City, IA 51104, USA; ^3^Pediatric Infectious Diseases, Department of Pediatrics, St. Joseph's Children's Hospital, 703 Main Street, Paterson, New Jersey 07503, USA; ^4^Department of Pediatrics, St. Joseph's Children's Hospital, 703 Main Street, Paterson, New Jersey 07503, USA; ^5^Pediatric Intensive Care, Department of Pediatrics, St. Joseph's Children's Hospital, 703 Main Street, Paterson, New Jersey 07503, USA

## Abstract

Enteritis as the only manifestation of novel coronavirus disease 2019 (COVID-19) in adolescents without features of multisystem inflammatory syndrome in children (MIS-C) or a prior history of inflammatory bowel disease (IBD) has not been described. We report two adolescent patients (a 14-year-old male and a 20-year-old pregnant female) presenting to tertiary-care centers in the United States with severe enteritis as the only manifestation of COVID-19 caused by severe acute respiratory syndrome coronavirus 2 (SARS-CoV-2) infection. The patients were hospitalized with acute abdominal pain and gastrointestinal (GI) bleeding, with no evidence of MIS-C, and were previously healthy with no history of IBD. The patients' nasopharyngeal swabs were positive for SARS-CoV-2 infection by reverse transcription polymerase chain reaction (RT-PCR), and testing for other infectious etiologies was negative. Both patients received intravenous corticosteroids and recovered without short-term complications. None of the patients died. This report highlights the need for keeping a high index of suspicion for SARS-CoV-2 infection in adolescents presenting solely with gastrointestinal manifestations, in the absence of respiratory symptoms or multisystem involvement, for prompt recognition and timely management.

## 1. Case 1

**Figure 1:**
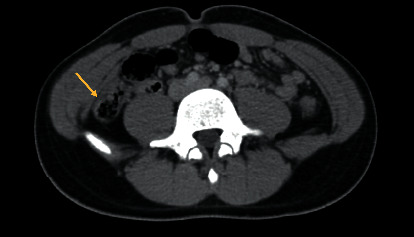
Computed tomography (CT) scan of the abdomen and pelvis with intravenous and oral contrast showing a thickened loop of bowel (arrow) in the right lower quadrant with ascites likely webbed into the colovesical pouch of the right paracolic gutter suggestive of ileitis.

A 14-year-old male with no significant past medical history presented to the ED (emergency department) in May 2020 with acute onset of severe epigastric and lower abdominal pain, nausea, and one episode of vomiting. In the ER, he had a temperature of 36.9°C (98.4°F), heart rate of 99 beats per minute, respiratory rate of 24 breaths per minute, and oxygen saturation of 99% on ambient air. The pain was described as 10/10 in intensity and nonradiating. Abdominal examination demonstrated a nondistended abdomen with guarding and tenderness in the epigastric region and below the umbilicus. Chest and abdominal radiographs were unremarkable for pathology. The patient's nasopharyngeal swab for SARS-CoV-2 RT-PCR was positive. Due to increased abdominal pain and rebound tenderness, computed tomography (CT) scan of the abdomen and pelvis was performed that showed a very thickened loop of bowel in the right lower quadrant with ascites, suggestive of enteritis/ileitis (Figure 1). The patient developed worsening abdominal pain and had one episode of vomiting containing frank blood and was admitted to the pediatric intensive care unit with necessary infection-control measures. Given the presumptive diagnosis of SARS-CoV-2 ileitis, the recommended regimen at that time was initiated: vitamin C, vitamin D, thiamine, zinc, melatonin, hydroxychloroquine, and famotidine. His complete blood count, liver function tests, and coagulation profile including fibrinogen level, ESR (erythrocyte sedimentation rate), CRP (C-reactive protein), D-dimers, ferritin, troponin, B-type natriuretic peptide (BNP), lactic acid, albumin, and CK (creatine kinase) levels were within normal limits. On the second day of admission, an increased total bilirubin level of 4.3 mg/dL (normal: 0.1–1.2 mg/dL) was noted with a direct bilirubin of 0.6 mg/dL (normal: <0.3 mg/dL). Hydroxychloroquine was discontinued due to hyperbilirubinemia, and cefoxitin, metronidazole, and methylprednisolone (at 40 mg/day) for ileitis were initiated. Stool testing for pathogens including bacterial cultures, *Clostridium difficile* PCR, and *Helicobacter pylori* fecal antigen were negative. The patient's clinical status gradually improved, and after 6 days of hospitalization, the patient was discharged home with a tapered prednisolone schedule. He has remained asymptomatic since hospital discharge.

## 2. Case 2

A 20-year-old previously healthy G1P0 female at 22 weeks' gestation presented to the ED with 2 days of severe lower abdominal pain, vomiting, reduced appetite, and diarrhea, with blood in stools for 1 day. She was admitted to the intensive care unit (ICU) for monitoring. At admission, she had a temperature of 36.8°C, heart rate of 110 beats per minute, respiratory rate of 22 breaths per minute, oxygen saturation of 100% on room air, and blood pressure of 130/84 mmHg. The patient was noted to have diffuse lower abdominal tenderness below the umbilicus with no rebound and had red blood mixed with loose stools. On laboratory evaluation, her inflammatory markers, liver function tests, blood cell counts, coagulation profile, D-dimers, ferritin, and albumin levels were normal. The patient had a negative respiratory viral panel (RVP) and a positive nasopharyngeal SARS-CoV-2 RT-PCR. Abdominal ultrasound was consistent with stated gestational age and was otherwise unremarkable. Stool cultures and 2-step stool testing for PCR and antigen for *Clostridium difficile* were negative. The patient was diagnosed with acute hemorrhagic colitis associated with SARS-CoV-2 infection and received intravenous fluids and intravenous dexamethasone (at 6 mg/day). Broad-spectrum antibiotics (vancomycin and piperacillin/tazobactam) were discontinued after 48 hours when cultures returned negative. Gradually, the abdominal pain and diarrhea improved with no recurrence of bleeding after day 2 of hospitalization. In view of the clinical improvement, it was decided to defer endoscopy. The patient was discharged and gradually weaned off steroids, with no recurrence of symptoms noted upon outpatient follow-up.

## 3. Discussion

To our knowledge, this is the first report of severe enteritis as the only manifestation of SARS-CoV-2 infection in two adolescent patients hospitalized at tertiary-care centers in Northeastern and Midwestern United States. Both patients, one of whom was pregnant, were aged less than 21 years of age, with no previous history of inflammatory bowel disease (IBD), and presented with gastrointestinal inflammation (ileitis and hemorrhagic colitis) as the presenting and the only manifestation of COVID-19, without any evidence of multisystem inflammatory syndrome in children (MIS-C).

In patients less than <21 years of age, gastrointestinal (GI) symptoms have been recently recognized in association with the novel syndrome labeled as MIS-C associated with SARS-CoV-2 infection [1]. Case definition for MIS-C has been provided by the CDC [2]. All children (aged <21 years) in the first-described cohort of MIS-C in United Kingdom in late April had GI symptoms, of whom 1 died [3, 4]. A recent study described similar experience in the United States, where all children with MIS-C had GI manifestations with markedly elevated inflammatory markers and rash as a prominent feature in the majority. Our patients did not have elevated biomarkers of systemic inflammation or multisystem involvement and thus did not meet criteria for MIS-C [2]. Moreover, no rashes/other signs and symptoms were seen in our patients, with no respiratory/cardiovascular dysfunction, unlike previously described cases of MIS-C [5, 6]. Also with MIS-C, multiorgan/system dysfunction is usual involving 2 or more systems [2], but in our patients, only gastrointestinal system was involved with no other organ-system dysfunction.

Patients with MIS-C have had positive antibody testing with or without positive RT-PCR for SARS-CoV-2, indicating that MIS-C is possibly a postinfectious immune-mediated phenomenon [2, 3, 5], while both of our patients had a positive SARS-CoV-2 nucleic acid testing by RT-PCR likely suggestive of active infection, indicating that enteritis was the presenting feature of acute SARS-CoV-2 infection in our patients.

Our patients received intravenous corticosteroids and recovered without any short-term complications and none of the patients died, unlike instances of MIS-C where mortality has been reported [3].

Adult patients with COVID-19 commonly present with respiratory symptoms and report GI symptoms in <10–15% of cases [6, 7]. Moreover, in adults with preexisting IBD, respiratory complications as well as IBD flares have been reported with SARS-CoV-2 infection [8]. However, in the adolescent age group, severe enteritis as the sole presenting feature of SARS-CoV-2 with no prior episodes/diagnosis of IBD has not been reported to date. In contrast to the usual presentation in older adults, pulmonary involvement was not noted in our patients.

Also, GI inflammation as the only manifestation of COVID-19 with pregnancy with no prior history of IBD has not been previously described. Flaring of symptoms in an adult patient with preexisting ulcerative colitis during pregnancy with COVID-19 was recently reported where the outcome was unfortunately fetal loss [9], unlike our adolescent patient, who was previously healthy with no prior history of gastrointestinal disease or IBD, with more advanced pregnancy at >20 weeks and a favorable outcome with preservation of pregnancy.

GI manifestations as the only presenting features of SARS-CoV-2 infection are rare, and prompt recognition is essential to institute optimal therapy as well as infection prevention measures. Long-term follow-up to monitor recurrent symptoms or other gastrointestinal complications is imperative.

## 4. Conclusion

This report, to our knowledge, is the first to describe severe enteritis as the only manifestation of SARS-CoV-2 infection in two previously healthy adolescent patients, one of whom was pregnant, with no characteristics of MIS-C or previous history of inflammatory bowel disease. As we are encountering novel manifestations of COVID-19, it is important to recognize nonrespiratory symptoms as the presenting features of SARS-CoV-2 infection. Further studies are needed for understanding of long-term complications and prognosis of this novel infection.

## Data Availability

The data used to support the findings of this study are included within the article.
